# Radiomics AI prediction for head and neck squamous cell carcinoma (HNSCC) prognosis and recurrence with target volume approach

**DOI:** 10.1259/bjro.20200073

**Published:** 2021-07-05

**Authors:** Tang FH, Chu CYW, Cheung EYW

**Affiliations:** 1 School of Medical and Health Sciences, Tung Wah College, Hong Kong, Hong Kong

## Abstract

**Objectives:**

To evaluate the performance of radiomics features extracted from planning target volume (PTV) and gross tumor volume (GTV) in the prediction of the death prognosis and cancer recurrence rate for head and neck squamous cell carcinoma (HNSCC).

**Methods:**

188 HNSCC patients’ planning CT images with radiotherapy structures sets were acquired from Cancer Imaging Archive (TCIA). The 3D slicer (v. 4.10.2) with the PyRadiomics extension (Computational Imaging and Bioinformatics Lab, Harvard medical School) was used to extract radiomics features from the radiotherapy planning images. An in-house developed deep learning artificial neural networks (DL-ANN) model was used to predict death prognosis and cancer recurrence rate based on the features extracted from GTV and PTV of the CT images.

**Results:**

The PTV radiomics features with DL-ANN model could achieve 77.7% accuracy with overall AUC equal to 0.934 and 0.932 when predicting HNSCC-related death prognosis and cancer recurrence respectively. Furthermore, the DL-ANN model can achieve an accuracy of 74.3% with AUC equal to 0.947 and 0.956 for the HNSCC-related death prognosis and cancer recurrence respectively using GTV features.

**Conclusion:**

Using both GTV and PTV radiomics features in the DL-ANN model, can aid in predicting HNSCC-related death prognosis and cancer recurrence. Clinicians may find it helpful in formulating different treatment regimens and facilitate personized medicine based on the predicted outcome when performing GTV and PTV delineation.

**Advances in knowledge:**

Radiomics features of GTV and PTV are reliable prognosis and recurrence predicting tools, which may help clinicians in GTV and PTV delineation to facilitate delivery of personalized treatment.

## Introduction

Head and neck squamous cell carcinoma (HNSCC) is the sixth most common cancer worldwide, accounts for approximately 550,000 new cases annually. In the United States, approximately 63,000 head and neck cancers being diagnosed every year. The standard treatment included surgery and post-operative radiotherapy as adjuvant, or concurrent chemoirradiation for unresectable cases.^1–3^ However, the 5 year overall survival was over 90% for early stage to below 50% for late stage.^4^ In all cases, locoregional control failure is the major cause of distant metastasis, which increase the risk of cancer recurrence and have poor prognosis.

With the rise of the artificial intelligence (AI) community in the last decade, medical applications of AI are becoming a more popular topic. Not only can it reduce potential errors and increase efficiency, but it also can help disclose important details of diseases to physicians. Recently, myriads of work^5–8^ have focused on the use of machine learning in producing prediction models by detecting possible patterns of data, which may improve clinical decision-making processes. Previous studies^9,10^ have also suggested that machine learning can potentially enhance the workflow management of radiation oncology.

Head and neck cancer can be treated with a wide range of treatment modalities, depending on regional anatomy and intratumoral heterogeneity. It also presents a great therapeutic challenge as there is a minute scale of critical structures and the variable anatomic changes during treatments. With the novel development of AI and new imaging data extraction techniques, they are hoped to be used to generate a clinical toolset for decision-making processes. Studies^11,12^ indicated that AI was used in tumor and organs at risk (OARs) segmentation, improved atlas-based contouring for lungs and spinal cords in lung cancers, as well as automatic segmentation of clinical target volume and OARs for rectal cancers. These applications may potentially improve the contouring in complicated head and neck cancer.

### Radiomics

Radiomics characterizes the phenotypes of tumors by extracting some high-dimensional data from clinical imaging.^13^ These are quantitative features that can provide specific information on tumor heterogeneity, texture, intensity, and morphology information, which could infer the tumor histology, grades, metabolism and even patient survival.^14^ This quantitative image feature approach is a breakthrough in clinical practice as it can potentially indicate prognosis by means of noninvasive, fast and cost-efficient procedures.

In addition, other radiogenomic studies have revealed the underlying gene-expression profiles of cancer patients, which may entail supplementary prognostic factors. More importantly, these quantitative features are sight to aid in the development of personalized medicine.^2^ Traditionally, patients are subjected to invasive biopsy procedures to determine the tumor histology and oncologic diagnosis. Treatment approaches are also primarily depending on the tumor/node/metastasis (TNM) staging system that is dependent on the resectability and grades of tumors. The application of radiomics data could relieve the sole dependence on invasive procedures, yet, generate reliable prognostic and biologic information. Aerts et al^2^ depicted clinical impact of radiomics in both HNSCC and non-small cell lung cancer (NSCLC).^2^ There were survival prediction models based on different image biomarkers^15^ and Human Papilloma Virus-16 (HPV-16) status.^16^ Equally important, the biomarkers within the tumor and its heterogeneity were suggested to correlate with the resistance or sensitivity to radiation.^17^ Several studies investigated and reviewed the intricate relationship between the lung cancer molecular makeup and radioresistance mechanism^18,19^ ; while others addressed the possible associations between the genomic heterogeneity and likelihood of metastasis based on MRI model.^20^

Initiatives in machine learning have been used to create prediction models, such as penalized logistical regression, artificial neural networks (ANNs), Bayesian networks (BNs), decision trees (DTs) and support vector machines (SVMs). High accuracy of classifications has been reported in studies based on SVM, which predicted the survival and recurrence of patients with oral cancer, breast cancer and cervical cancer.^21–23^

Segmentation is an imperative process for the radiomics analysis. In radiotherapy treatment, the gross tumor volume (GTV) defines the position and extent of gross tumor. The planning target volume (PTV), defines the position of GTV, potential microscopic tumor spread and margin for daily setup uncertainties, allows for organ motions and intertreatment variation during treatment delivery. Radiotherapy planning must consider the radiation dose to critical normal tissue structures (OARs) to ensure that they receive a safe dose to preserve their function.^24^ A balance between homogeneous high dose to GTV and PTV as well as minimize dose to OAR is always an important issue in radiotherapy planning. GTV and PTV delineations are major steps to determine the success of the radiotherapy treatment. GTV and PTV are ‘must have’ items in the radiotherapy treatment planning CT images. In this study, we investigated the role of radiomics features of PTV and GTV, whether they can predict the treatment outcome including prognosis and recurrence rate accurately.

## Methods

### Patient data

The data were retrieved from a publicly available database, the Cancer Imaging Archive (TCIA) (*Data from Head and Neck Cancer CT Atlas*).^1^ Patients who were treated with radiotherapy for HNSCC, with curative-intent radiation therapy (RT) were collected from 1 Oct 2003 to 31 Aug 2013 in a single center. All patients were presented at a multidisciplinary tumor board for treatment recommendations. Diseases were staged per the American Joint Committee on cancer using the TNM system (AJCC-TMN).^25^ HNSCC standard treatment was offered depending on the site and stage of the tumor, including primary surgery, single-modality RT (66–70 Gy), or concurrent RT (66–72 Gy). For patients who underwent primary surgery, post-operative RT or concurrent chemotherapy was offered. Induction chemotherapy was offered to patient with high risk, advanced stage in T and N at the discretion of oncologist. The primary gross tumor volume (GTV) and the primary PTV was contoured by two radiation oncologists in the center where the images collected. GTV and PTV of lymph nodes were also contoured, but they were excluded in this study.

Patients had planning CT images available, tumor stage was not Tx (primary tumor could not be assessed), T0 (no evidence of primary tumor) or Tis (carcinoma *in situ*), and the nodal stage was not Nx (regional lymph node could not be assessed) were included in this study. Patient who received radiotherapy treatment prior to this study, had recurrence of HNSCC, or patients whose GTV or PTV on CT images was affected by artifacts were excluded from this study.

There were 215 patients’ CT data set were acquired. 27 patients were excluded from this data set due to missing data elements or issues related to importing data into the radiomics software.

Also, patient demographic data, including age, gender, diagnosis (carcinoma of base of tongue, carcinoma of supraglottis, carcinoma of tonsil, and other head & neck cancers), staging at diagnosis (I, II, III, IVA, IVB), smoking status at diagnosis (smoking or non-smoking) and treatment modalities received before radiotherapy (chemotherapy only, surgery only, both surgery & chemotherapy) were collected for further analysis.

The study was approved by the institutional research ethics committee of the Tung Wah College (REC2019031). Written informed consent was obtained from all study participants by the data collection institution.

### Workflow of this study

**Figure 1.**
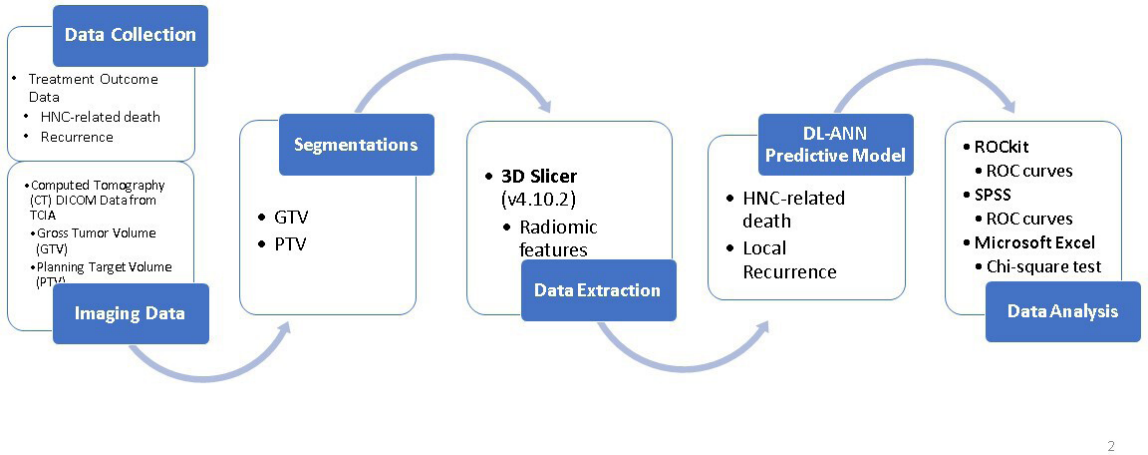
Diagram to show the workflow of this study. GTV, gross tumor volume;PTV, planning target volume; HNC, head and neck cancer.

As an initial step, CT images were retrieved from TCIA along with contoured treatment volumes and different clinical data. Then, radiomics data were extracted and inputted into the DL- ANN predictive model using deep-learning. Further statistical analyses were done on the results (Figure 1):

### Feature extraction

The 3D slicer (v. 4.10.2) with the PyRadiomics extension (Computational Imaging and Bioinformatics Lab, Harvard medical School)^2^ was used to extract radiomics data from the planning CT images. The primary gross tumor volume (GTV) and PTV contoured were used for segmentations. 107 features were extracted as signature values to create a predictive model. The radiomics features consists of the tumor’s shape (*n* = 14), gray level dependence matrix (*n* = 14), gray level co-occurrence matrix (*n* = 24), first-order statistics (*n* = 18), gray level run length matrix (*n* = 16), gray level size zone matrix (*n* = 16) and neighboring gray tone difference matrix features (*n* = 5) (see :https://pyradiomics.readthedocs.io/en/latest/features.html for the features details). A total of 107 radiomics features were extracted per patient from the planning CT images. These features were used as input data to the ANN model for death prognosis and cancer recurrence prediction.

### Artificial neural networks and machine learning

ANNs are dynamic computational models that mimic the human brain to acquire knowledge and learn to process information,^26^ utilized for modeling, pattern recognition, classification and multivariate data analysis.^27^ The underlying mechanism consists of multiple intermediate layers (also known as hidden layers), representing the interconnected neurons contained in the biological neural network. The DL-ANNs model in the current study was in-house developed by investigator (Tang) implemented by MATLAB (MathWorks^®^, v. 2018a) and comprised of four hidden layers.

#### Architecture of the ANNs models

**Figure 2.**
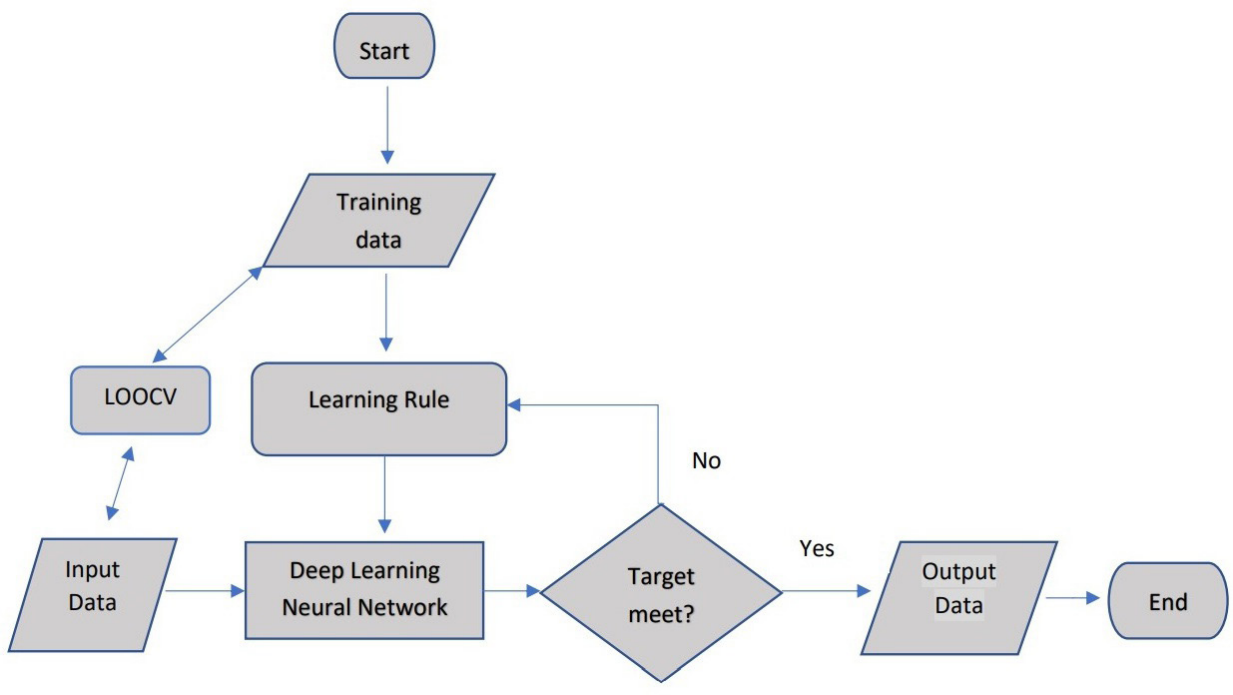
The Architecture of DL-ANN Model used in this study. DL-ANN, deep learning-artificial neural network.

The architecture of the model is described in Figure 2.

Training data: the 107 radiomics features extracted from 90 cases for death prognosis prediction, 100 cases for cancer recurrence prediction were served as input training data.

Learning rule: the use of the Rectified Linear Unit (ReLU) function as the activation function. This function gives 0 if the inputs are negative; while it gives 1 if the inputs are positive and allows the input values to go to next step.

Input data and leave-one-out cross-validation (LOOCV): the mechanism was to use all data as training but leave one for validation each time. The process was repeated until all data were used as training–validation combination.

Deep learning ANN (DL-ANN): it is a network with three hidden layers, 107 inputs and 1 binary (0: “disease free” or 1 “abnormal, patient death or cancer recurrence“) outputs.

Target: error was set as less than 0.01, with 1000 epochs of training

Output: corresponding cases with “0” or “1”.

### Training and validation

All data were used as training and validation cohort simultaneously using LOOCV.^28^ The mechanism is to input all data as training set but leave one as validation each time. The process was repeated until all data were used as training–validation combination. This is a particularly effective validation test for models with smaller sample size as the training samples of each time would resemble each other.

The final outcome was presented as 0 and 1. In the death prognosis prediction, 0 indicated as patient survives after 5 years or died of non-HNSCC-related disease, while one indicated as patient died within 5 years after diagnosis. In the cancer recurrence prediction, 0 indicated no recurrence being detected within 5 years after diagnosis, while 1 indicated local recurrence or distant metastasis was found within 5 years after diagnosis.

It should be noted that in cancer death prognosis, the survival rate is determined at a specific time point, such as 5-years or 10-years after diagnosis.^29^ This is established to accommodate—different views towards cancer survivorship. A standardized cancer survival rate improved the objectivity in comparing the prognosis among cancers, as well as the effectiveness of different treatment modalities. 5-year survival rate and 10-year survival rate has been used since mid-1970s. Most researchers and medical professionals classified patients as—“cancer survivors” when they—had survived 5 years after their last treatment received. They found that it is a time when the risk of a recurrent cancer had diminished substantially.^30,31^ For this reason, the 5-year survival was chosen in death prognosis prediction—in this study.

The algorithm performance analysis was evaluated using concordance statistics (c-index), which was also known as the area under the ROC curve (AUC). ROCkit (1995) and SPSS (v. 26.0) were used to generate ROC curve and AUC or c-index.

## Result

### Demographics of cohort

In this study, 188 patients CT data set—with GTV and PTV were collected. For further analysis in groups, PTV by diagnosis: Ca Base of Tongue (*n* = 72), Ca Supraglottis (*n* = 12), Ca Tonsil (*n* = 61), Ca others (*n* = 43). By staging: Stage I (*n* = 4), Stage II (*n* = 4), Stage III (*n* = 24), Stage IVa (*n* = 138) and Stage IVb (*n* = 18). By smoking status: non-smokers (*n* = 131) and smokers (*n* = 57). 25 patients were not analyzed in the GTV due to no contoured GTV. 163 cases with GTV were collected. Moreover, there were 96 patients who received chemotherapy only, 47 patients who received surgery only, and 20 cases received both chemotherapy and surgery prior to radiotherapy. The details are listed in Table 1

**Table 1.** Patient demographic, tumor characteristics and clinical data

**Patient and tumor characteristics (PTV *n* = 188**)	**Data**
**Age range**	24–91
**Sex**	
Female	21
Male	167
**Staging**	
Stage I	4
Stage II	4
Stage III	24
Stage IVA	138
Stage IVB	18
**Diagnosis**	
Ca BOT	79
Ca supraglottis	18
Ca tonsil	67
Ca others	51
**Smoking status**	
Smoker	57
Non-smoker	131
**Patient and tumor characteristics (GTV *n* = 163)**	**Data**
**Treatment received before RT**	
Chemotherapy only	96
Surgery only	47
Chemotherapy & surgery	20

BOT, Base of Tongue; GTV, gross tumor volume; RT, radiation therapy.

### Prediction of HNSCC-related death prognosis

Using the PTV features to predict death prognosis, the ANN model achieved an accuracy of 77.7%, with sensitivity of 95.6% and specificity of 72.0% (AUC = 0.9250) for all 188 patients.

When using the GTV features to predict death prognosis, the DL-ANN model achieved an accuracy of 85.9% (AUC = 0.9460), with sensitivity of 84.2% and specificity of 86.4%in for all 163 patients.

**Figure 3.**
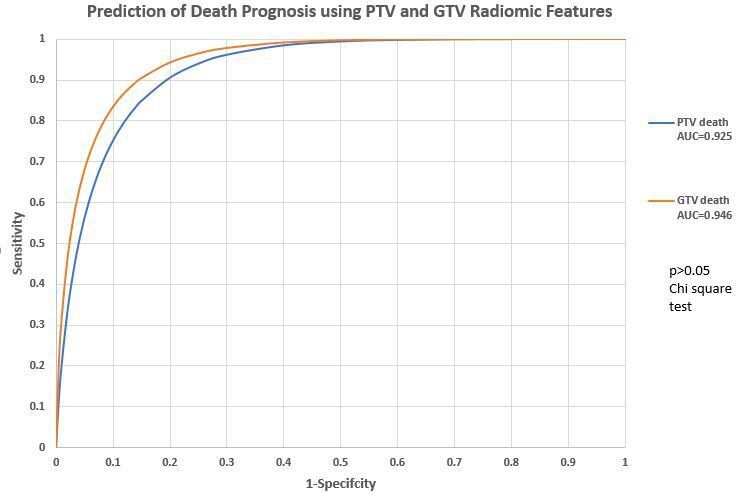
Prediction of death prognosis using PTV and GTV radiomics features. GTV, gross tumor volume;PTV, planning target volume.

The ROC analysis indicated that there was no significant difference between using PTV and GTV radiomics features to predict death prognosis and cancer recurrence (*p* > 0.05). The details are listed in Figure 3, Table 2.

**Table 2.** Performance of DL-ANN model for head and neck cancer to predict death prognosis and cancer recurrence

		Accuracy	Sensitivity	Specificity	Negative case	Positive case	Case tested
**PTV**	Death prognosis	0.7766	0.9556	0.7203	143	45	188
	Cancer recurrence	0.7433	0.9672	0.6349	126	61	187*
**GTV**	Death prognosis	0.8589	0.8421	0.864	125	38	163
	Cancer recurrence	0.7239	0.9623	0.6091	110	53	163

DL-ANN, deep learning artificial neural network; GTV, gross tumor volume; PTV, planning target volume.

* 1* one case is omitted due to data error

### Prediction of cancer recurrence

Of the 187 cases^1^ with PTV radiomics features to predict cancer recurrence, our DL-ANN model was able to obtain an accuracy of 74.3% with sensitivity of 96.7% and specificity of 63.5% (AUC = 0.93).

Using GTV radiomics features, the DL-ANN model can obtain an accuracy of 72.4% (sensitivity = 96.2%, specificity = 60.9%, AUC = 0.96).

**Figure 4.**
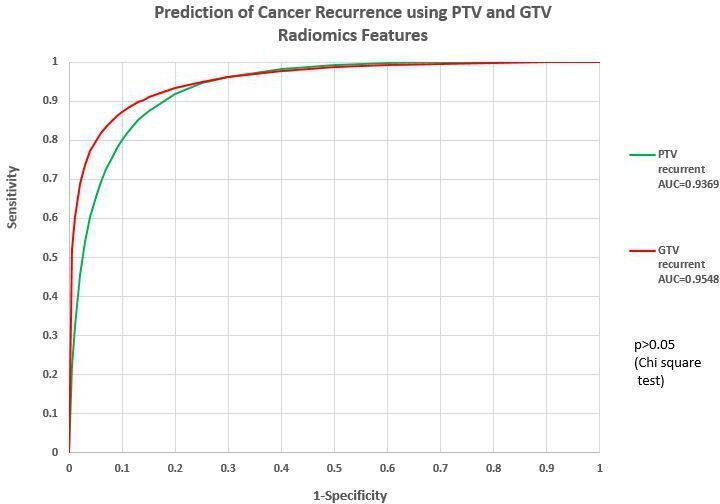
Prediction of cancer recurrence using PTV and GTV radiomics features. GTV, gross tumor volume;PTV, planning target volume.

The ROC analysis indicated that there was no significant difference between using—PTV and GTV radiomics features to predict cancer recurrence (*p* > 0.05). The details are listed in Figure 4, Table 2.

### Prediction capability of PTV radiomics features

**Figure 5.**
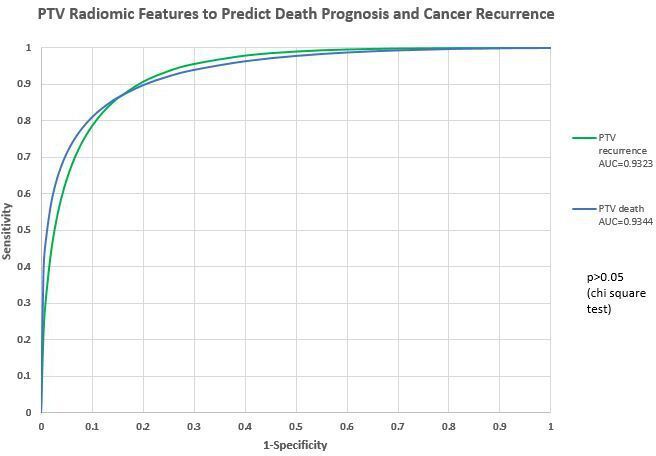
Prediction capability of PTV radiomics features. AUC, area under the curve; PTV, planning targetvolume.

There was no significant difference between prediction of death prognosis and cancer recurrence using PTV radiomics features (χ^2^ test, *p* > 0.05). The details are listed in Figure 5, Table 3.

**Table 3.** ROC study comparison: PTV and GTV radiomics features to predict death prognosis and cancer recurrence

	Death prognosis	Cancer recurrence	χ^2^ test
AUC (PTV)	0.934	0.932	*p* = 0.4071 (>0.05)
AUC (GTV)	0.947	0.956	*p* = 0.5296 (>0.05)

AUC, area under the curve; GTV, gross tumor volume; PTV, planning target volume; ROC, receiver operating characteristic.

### Prediction capability of GTV radiomics features

**Figure 6.**
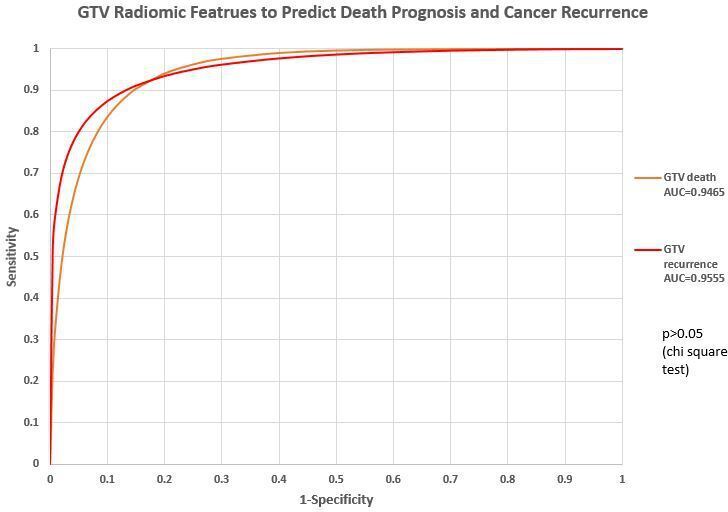
Prediction capability of PTV radiomics features. AUC, area under the curve; GTV, gross tumor volume; PTV, planning targetvolume.

When using ROC analysis, there was no significant difference between death prognosis and cancer recurrence prediction using GTV radiomics features (χ^2^ test, *p* > 0.05) The details are listed in Figure 6, Table 4 .

**Table 4.** ROC study comparison: death prognosis and cancer recurrence prediction by PTV and GTV radiomics features

	GTV	PTV	χ^2^ test
AUC (Death prognosis)	0.946	0.9250	*p* = 0.8955 (>0.05)
AUC (Cancer recurrence)	0.9555	0.9369	*p* = 0.6415 (>0.05)

## Discussion

This study evaluated the prognostic value of planning CT in HNSCC regarding GTV- and PTV-based radiomics features. The performance of the model was validated using LOOCV. It appears that our deep learning models are able to generate a promising prediction for death prognosis and cancer recurrence based on the GTV and PTV radiomics data.

### Treatment decision-making process with aids of GTV features

The traditional way of predicting one’s prognostic outcome is based on TNM staging system. It is regarded as a valid tool to evaluate treatment approaches based on the tumor size, histology, local invasion, lymphatic spread, and metastasis. In HNSCC, locoregional tumor control closely related to the survival.^32^ In recent years, advances in technology have enabled analyses of medical images based on general tumor phenotypic and genomic features. Shakir et al study showed that radiomics features of HNC with neural network showed promising result in tumor histology classification.^33^ Other studies showed correlations between clinical outcomes and radiomics features in various types of cancer.^34,35^ Subsequently, these technique could play a complimentary role, along with the TNM staging system, in the treatment decision-making process.

In this study, the predictive model was trained to predict death prognosis and cancer recurrence. For the death prognosis prediction, our model with GTV radiomics features was able to yield a promising classification result (Accuracy = 85.9%, AUC = 0.947). It was particularly significant as each subgroup had a relatively large sample size. Similar observation appeared in the cancer recurrence prediction with GTV radiomics data sets (accuracy = 72.4%, AUC = 0.956). There is an improvement when compared with the study by Bryce et al, where the authors used clinical factors like nodal stage and tumor size, stage, resectability and hemoglobin level to predict a 2-year survival.^36^ Their model yielded an AUC of 0.67, while the specificity and sensitivity were 72 and 70%, respectively. As technology is getting advanced, better models are expected to improve the predictive accuracy to support treatment decision.

### Death prognosis and cancer recurrence predictions based on PTV features

In this study, the PTV was used for image segmentation for radiomics data extraction. In fact, PTV is a geometric boundary to ensure the radiotherapy prescription dose is actually delivered to the clinical target volume (CTV) and it is a volume related to the isocenter of the linear accelerator rather than to the anatomy of the patient. Thus, it is a more practical treatment volume for use in radiotherapy planning and treatment procedure.^24^ Our study filled an important knowledge gap in similar studies^13,20,37^ where treatment margins such as PTV were not incorporated in predictions of treatment outcomes. Reduction of CTV–PTV margin has long been discussed to minimize the radiation-related toxicity. Previous studies proved that reduction in the CTV–PTV margin from 5 to 3 mm with daily CBCT-guided radiotherapy reduced the radiation toxicity without compromised the treatment outcome.^38,39^ Our result may improve the application through including the PTV radiomics features into the delineation criteria, instead of considering the geometrical reduction in PTV margin only.

In consideration of survival prediction, Yu et al^16^ study developed several predictive models to classify HPV-16 status using radiomics data.^16^ Their prediction model of 5-year survival based on logistic regression had an AUC of 0.67. It appears our model using DL-ANNs yielded more promising result (AUC = 0.947, GTV segmentation). On the other hand, Vallieres et al 2015 study used radiomics model for prediction of lung metastasis from PET and MRI texture features,^40^ their best performance AUC was 0.984, which was comparable with our study for cancer recurrence predictions (AUC = 0.932 for using PTV, AUC = 0.956 using GTV).

### Limitations

The analysis was based on single-center data, the proposed model should be further validated by an external cohort to confirm its application in planning CT collected by different scanners. Also, both PTV and GTV radiomics were used to predict death prognosis and cancer recurrence, while some groups with small sample size were excluded. Furthermore, GTV was not available in some cases which reduce the sample size for GTV. Larger data set was recommended for future studies to improve the model accuracy. Also, including PTV and GTV radiomics features from other imaging modalities, *e.g.* PET/CT and MRI may help to develop a more comprehensive model.

## Conclusion

This study sought to assess the use of radiomics and ANN predictive models to predict different treatment outcomes, including death prognosis and cancer recurrence. The resulting model was able to yield promising death prognosis and cancer recurrence prediction based on the GTV and PTV radiomics features.

The significant outcome of this study exhibits good predictive abilities of death prognosis and cancer recurrence. The enhancement of accuracy provided insights on future models that may assist doctors in personalized medicine, facilitating them to determine which treatment modality, as well as the boundary of PTV may produce a better outcome for the patient. However, prior to implementing the model into clinical practice, they should be thoroughly assessed to prove their substantial benefits.

## Footnotes

* 1 case was omitted from the 188 cases collected due to data error
